# Natural Inspired Intelligent Visual Computing and Its Application to Viticulture

**DOI:** 10.3390/s17061186

**Published:** 2017-05-23

**Authors:** Li Minn Ang, Kah Phooi Seng, Feng Lu Ge

**Affiliations:** 1 School of Computing & Mathematics, Charles Sturt University, Wagga Wagga 2678, Australia; lang@csu.edu.au; 2 National Grape and Wine Industries Centre, Wagga Wagga 2678, Australia; 3 CM3 Research Centre, Charles Sturt University, Bathurst 2795, Australia; fge@csu.edu.au

**Keywords:** natural inspired computing, intelligent system, artificial immune system, visual information processing, viticulture applications

## Abstract

This paper presents an investigation of natural inspired intelligent computing and its corresponding application towards visual information processing systems for viticulture. The paper has three contributions: (1) a review of visual information processing applications for viticulture; (2) the development of natural inspired computing algorithms based on artificial immune system (AIS) techniques for grape berry detection; and (3) the application of the developed algorithms towards real-world grape berry images captured in natural conditions from vineyards in Australia. The AIS algorithms in (2) were developed based on a nature-inspired clonal selection algorithm (CSA) which is able to detect the arcs in the berry images with precision, based on a fitness model. The arcs detected are then extended to perform the multiple arcs and ring detectors information processing for the berry detection application. The performance of the developed algorithms were compared with traditional image processing algorithms like the circular Hough transform (CHT) and other well-known circle detection methods. The proposed AIS approach gave a Fscore of 0.71 compared with Fscores of 0.28 and 0.30 for the CHT and a parameter-free circle detection technique (RPCD) respectively.

## 1. Introduction

Natural Computing refers to human-designed computational processes observed and inspired by nature. It can be broadly divided into three main branches: (1) the first and main branch is computing inspired by nature. Inspiration from nature is taken into account to develop computational tools or algorithms to solve complex problems; (2) the second branch is the simulation and emulation of nature by means of computing aimed at creating patterns and behaviors to increase the understanding of nature and give insights about computer models; and (3) the third branch is the computing with natural materials through the use of novel materials to perform computation. The field of natural computing includes evolutionary algorithms, artificial neural networks and immune systems, DNA, molecular and quantum computing. The use of computer vision and machine learning is increasingly popular for agriculture analytics (agri-analytics) applications. This paper has the objective to investigate the use and potential of natural and visual computing approaches and technologies for application in viticulture. The paper has three contributions: (1) a review of visual information processing applications for viticulture; (2) the development of natural inspired computing algorithms based on artificial immune system (AIS) techniques for grape berry detection; and (3) the application of the developed algorithms towards real-world grape berry images captured in natural conditions from vineyards in Australia. The aim of this paper is to be useful for researchers to get insights into this important area, motivate the development of practical solutions towards deployment in practical vineyards, and to give some contributions and show some potential for using nature-inspired computing in viticulture.

Artificial immune system (AIS) is one of the emerging members of the natural computation intelligence family. AIS are a class of computationally intelligent systems inspired by the principles and processes of the vertebrate immune system. AIS algorithms are modeled after the characteristics of learning and memory found in immune systems for use in problem-solving and optimization [1,2]. Research on AISs can be divided into three areas: (1) immune modeling; (2) theoretical AIS models; and (3) applied AIS algorithms. Immune modeling focuses on research work developing models and simulations of natural artificial immune systems. Theoretical AIS models aim at describing and the mathematical modeling, performance and complexity analysis of such algorithms. The research on the theoretical aspects has been centered on four major types of AIS algorithms: (1) negative selection; (2) artificial immune networks; (3) clonal selection; and (4) danger theory and dendritic cell algorithms.

AIS has been used for research in many applications areas [3,4,5,6]. The major applications of AIS can be generally grouped into the following four areas: (1) classification/clustering; (2) anomaly detection; (3) optimization; and (4) supervised/unsupervised learning. Despite many successful applications of AIS, it has not been widely used or applied to the field of agriculture or viticulture, although the use of computer vision and machine learning is increasingly becoming popular for agri-analytics applications. Some applications of visual computing in this field involve automatic leaf estimation, fruit harvesting, yield estimation, grape quality evaluation and grapevine variety identification. The research of visual computing in viticulture is not fully mature and there are several challenges to be addressed. Specifically, the potential of natural inspired computing such as AIS for applications in viticulture has not been exploited or explored. This paper presents a natural inspired computing approach using AIS techniques for a grape berry detection viticulture application. The AIS algorithms were developed based on a nature-inspired clonal selection algorithm which is able to detect the arcs in the berry images with precision, based on a fitness model.

**Figure 1:**
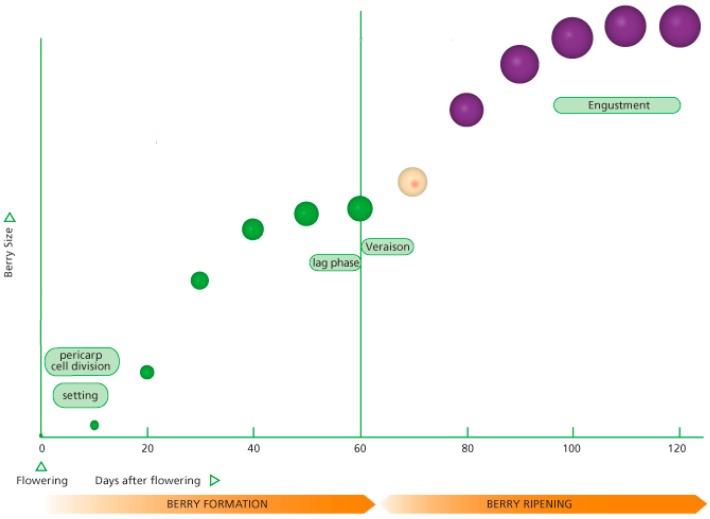
Growth curve for grape berry formation to ripening curve [7].

Figure 1 shows a typical growth berry formation to berry ripening curve [7]. The details for berry formation can be found in [7,8]. Initially after flowering, the grape berries increase in size rapidly for about 40 days. The growth of the berries then slows and plateaus until about day 60. This denotes the growth phase known as *veraison* which denotes the onset of ripening. The grape berries then begin to increase in volume again for about another 40 days. This phase of grape berry growth is known as *engustment* when the aromas and flavors of the grape intensify while the tannins and anthocyanin (color) develop. Grape growers and viticulturists aim to harvest during *engustment* because this stage allows for the aromatic potential of the wine. During the *engustment* phase, the grape berry volume increases to a maximum and then begins to decrease. A rule of thumb used by wine experts is to harvest the grapes when a decrease of about 10% from the maximum berry volume is detected from the sampled bunches.

Multi-ring detection has been a challenging problem in the field of visual computing for viticulture due to the variable sizes and non-ideal shapes of grape berries in images. This paper proposes a new multi-ring detection algorithm based on AIS to detect grape berries with good precision in captured images. Our approach uses two steps: (1) a stochastic search based on real-value artificial immune systems for detecting multiple arcs for berry images with good precision; and (2) a heuristic mechanism to extend the arcs detected to locate the visible circular berries and partially visible arcs. The stochastic search adapts the real-value clonal selection algorithm (CSA) towards the multiple arc detection problem. In contrast to the conventional binary-encoded AIS approach [9], the real value AIS utilizes a real valued vector representation for antibodies, and utilizes memory cells to store the arcs detected during the processing.

There are several advantages to employing a real valued representation compared to using a binary representation. First, only a small number of antigens are required. In immunology terms, an antigen is a substance that forces the immune system to produce antibodies against it. The initialization and mutation are all performed in the real-value domain. The disadvantage of a binary representation is that it requires several hundreds of antibodies to detect a few arc objects in the image. Second, by introducing memory cells to store the arcs detected during the iterations, the CSA saves computational time and memory space by providing all the detected solutions once the process completes. Third, the immunological process of antibodies destroying antigens is modeled by the operation of removing the arcs detected through the iterations. This reduces the number of antibodies for later iterations and gives further savings in computational time and storage space.

A common approach for circle and ring detection is to use the circular Hough transform (CHT) [10], which uses a projection between the image space and parameter space based on a circle definition function. The difficulty of the CHT is the huge amount of data in the parameter space during transformation. This not only takes a large amount of memory storage, but also makes finding the local maxima difficult to handle. For arc detection, the required memory storage becomes larger because arc objects require further parameters to be stored compared to circles. An approach used in practice is to quantize the parameter space (e.g., combining two-by-two accumulation pixels into a single accumulation bin). However, this would result in the quantized parameter space not being able to give the same resolution as the resolution in the original image space.

For our viticulture application, to detect berry arcs and/or circles reliably and with good precision in natural images, our approach focuses on identifying potential arc candidates from the edge map using the AIS. Our observation is that the berry edge map may lose some potential arc/circle pixels after pre-processing. Thus, our method focuses on detecting strong arc candidates first using the AIS. The arcs detected are then extended in the second step to perform the multiple arcs and ring detectors information processing for the berry detection viticulture application. The performance of the developed algorithms were compared with traditional image processing algorithms like the CHT and other well-known circle detection methods, and were found to give good results. The remainder of the paper is organized as follows: Section 2 reviews some existing visual information processing applications for viticulture applications, and also existing works on the AIS. Section 3 presents the proposed multi-arc AIS detection using the real-valued CSA representation. Experiments on real-world grape berry images captured from vineyards are presented in Section 4. Finally, some conclusions are given in Section 5.

## 2. Related Works

This section gives related works on two aspects: (i) Visual information processing applications for viticulture; and (ii) History and background works in AIS with emphasis on the approach used in this paper (i.e., clonal selection algorithms).

### 2.1. Visual Information Processing for Viticulture

Visual information processing (VIP) is becoming increasingly popular in agriculture and viticulture applications. Some examples for VIP in viticulture can be found in applications for yield estimation, disease detection, automated vineyard monitoring, and grape phenology.

#### 2.1.1. Yield Estimation

Yield estimation or forecasting is of critical importance to the wine industry, as it allows grape growers to more accurately invest in capital equipment, negotiate pricing, and develop marketing strategies [11,12]. Traditionally, forecasts are generated by manual sampling of bunch weights, grape size, and grape numbers. However, the manual process is labor intensive, expensive, and inaccurate. Berry parameters like berry number per cluster, berry size, and weight are useful parameters for yield estimation and appear as indicators for grape and wine quality in vineyards. The work by Nuske et al. [11] proposed an image processing approach to count grape berries to forecast the harvest yield. This is a challenging problem because of varying illuminations, shadows, occlusions and a lack of color contrast between white grape varieties within the similarly colored background. The authors used a radial symmetry transform using shape and texture features for the segmentation and detection task and showed that their approach could detect green berries against a green leaf background. To overcome the color and illumination variability found in daylight, the work by Font et al. [12] proposed a yield estimation method from the analysis of high-resolution images obtained using artificial illumination at night. The authors investigated five segmentation techniques (threshold segmentation, Mahalanobis distance segmentation, Bayesian classifier, direct three-dimensional histogram and linear color models). They showed that the use of controlled illumination at night combined with high-resolution images of the vineyard simplified the detection of grape clusters. Using a calibration procedure where the number of pixels corresponding to a cluster of grapes is computed and converted directly into a yield, the authors obtained an estimated yield error of 16%. The work by Dunn & Martin [13] proposed a small-scale yield estimation technique based on simple image color discrimination. This approach was evaluated on Shiraz post-*veraison* after color development and very close to harvest. We note that their method may not be applicable for the majority of real world examples where the berries appear over a background of similarly-colored leaves, as for white grape berry varieties and other varieties before *veraison* and the berry color formation. Recently, a work by Liu et al. [14] proposed a visual processing algorithm combining color and texture information and the use of a support vector machine (SVM) for berry detection. Their proposed segmentation algorithm contains three main steps: (1) image pre-processing; (2) training on a subset of images; and (3) segmentation on the test set. The authors used morphological operations applied in the HSV color space followed by a shape filter to remove incorrect grape bunches. The training set was formed from a group of human selected true bunches and the SVM was applied to segment bunches in the test set. Experiments performed on two varieties of red grapes demonstrated the average accuracy and recall of 87% and 90% respectively by using 5 fold validation.

#### 2.1.2. Disease Detection

The disease detection of grape berries in the field is a challenging problem due to four inter-related issues: (1) grape berries can show different signs and symptoms depending on the grape variety; (2) stage of development of the disease; (3) several diseases can be present at the same time; and (4) weather factors and nutrient deficiencies may also produce signs that are similar to the diseases. Important diseases for grapes to be identified include the powdery mildew, grape leaf roll virus, eutypa dieback, botrytis bunch rot and crown gall [15]. In Australia, wineries impose price penalties if 3% to 5% of the harvested crop have been affected by botrytis [16].

An early study by Weizheng et al. [17] in 2008 proposed to use image processing techniques in place of human inspection for grading plant diseases. Although the authors applied their research towards the gray leaf spot disease of soybean, their work demonstrated that the usage of image processing technology could eliminate the subjectivity of traditional human inspection methods and the corresponding human-induced errors. The work by Padol & Yadav [18] used a SVM classifier towards classifying two types of grape leaf diseases (downy mildew and powdery mildew). Their approach used K-means clustering for leaf area segmentation and performed SVM classification using color and texture features. The authors reported an accuracy rate of 93.33% and 83.33% for the detection of downy mildew and powdery mildew respectively for a dataset of 137 grape leaf images. A recent technique was proposed by Waghmare et al. for the detection and classification of grape plant diseases using local texture patterns and machine learning [19]. Their approach focused on the downy mildew and black rot diseases. To perform the leaf segmentation, the authors used a unique fractal based texture feature. Classification was performed using a multiclass SVM. The authors reported an accuracy of 96.6% based on advice from agricultural experts.

#### 2.1.3. Automated Vineyard Monitoring and Management

The work by Lloret et al. proposed a wireless sensor network (WSN) application for automated vineyard monitoring using imaging sensors [20]. The authors performed a testbed implementation of their automated system for a vineyard in Spain. Their WSN used sensor nodes which took images from the field and internally used image processing techniques to detect any unusual status in the grape leaves. This unusual symptom could be caused by a deficiency, pest, disease or another harmful agent. When a candidate detection is made, the sensor node sends a message to a sink node through the WSN to notify the farmer. Their WSN system used the IEEE 802.11 a/b/g/n standard, which allows connections from large distances in open air. Although their system was not able to distinguish between the various diseases, pests or other harmful agents, the authors remarked that a symptoms image database together with a neural classifier could be added to provide an accurate problem diagnosis.

#### 2.1.4. Grape Phenology

The grapevine phenology is a result of the complex interactions between the different plant genotypes and the environmental conditions. The grape phenolics are important determinants of wine quality. An early approach to estimate grape phenolics and color for entire vineyards utilized optical remote sensing technologies [21]. The work in [21] by Lamb et al. used high-resolution images which were acquired on three occasions during each of two consecutive growing seasons. The authors established a link between the physical descriptors of grapevine canopies from the remotely-sensed images and the subsequent measurements of the grape phenolics and color. The disadvantage of using remote sensing technologies is the high costs that are required. More recently, authors have proposed using digital imaging and computer vision techniques using cheaper ground-based cameras [22]. The work by Rodríguez-Pulido et al. [23] described a characterization of grape seeds and grape berries by digital image analysis and showed that the development of the grape could be visually determined by tracing its changes in size and color. The authors showed that for the red grape variety, the unripe berries are initially green, and then change to light pink, purple and black as they grow to full maturity. Another emerging trend for estimating grapevine phenology is using automated systems and mobile robotics technologies. The work by Kicherer et al. [24] illustrates this approach. The authors developed a phenotyping robot (*PHENObot*) consisting of a robotic platform, a multi-camera-system and a geo-information system in combination with an industrial computer. The *PHENObot* allows the image acquisition from 250 individual grapevines per hour for high throughput sampling of image data directly in the field. Together with this, the authors developed an automatic image analysis tool *BIVcolor* (Berries in Vineyards-color) to determine the precise phenotypic data of berry size and color within a large set of grape plants.

#### 2.1.5. Smart Vineyards and Knowledge Engineering Approaches for Viticulture

Some knowledge engineering approaches for viticulture have been demonstrated in the research works by Kamsu-Foguem et al. [25,26]. In these works, the authors proposed the Geoviticulture Multicriteria Climatic Classification (MCC) System and numerical and symbolic reasoning techniques with conceptual graphs for formal knowledge representation and visual reasoning using rules and queries for viticulture applications. Their system acts as a reference system for global viticulture and allows comparisons of viticultural climates for different regions of the world. The knowledge representation is symbolized with the conceptual graphs formalism and the reasoning mechanisms are based on graph operations. The authors illustrate the usefulness of their approach with a case study of Croatia republic and its broad characterizations of its main regional grape varieties. Another example of a smart vineyard for predicting diseases of vine is the PreDiVine Decision Support System reported by Prevostini et al. [27] which has been deployed in Switzerland and France in 2015. The aim of this support system is to aid in predicting the evolution of vineyard's pests and diseases and suggesting just in time targeted treatments.

### 2.2. AIS Models & Algorithms

Research in AIS accelerated by the end of the 1980s with the works by Farmer et al. [28] and Bersini & Varcia [29] on developing natural computing algorithms inspired by immune networks. Several models based on natural immune mechanisms have been developed to solve recognition and classification problems. The three major AIS models are immune network theory, the negative selection mechanism, and the clonal selection algorithm. In immune network theory, the immune system is modeled as maintaining an idiotypic network of interconnected B cells. Idiotypic network theory is based on the concept that lymphocytes are not isolated, but can communicate with each other through interaction among antibodies. Thus, the recognition of antigens is done at a system level by the sets of interactions of antigen-antibody as a network. The negative selection mechanism was proposed by Forrest et al. [30] and is based on the T cells’ ability to discriminate between self-nonself. During generation, T cells go through a censoring process in the thymus called negative selection where T cells that react against self-proteins are destroyed, while those that do not bind to self-proteins are allowed to maturate and circulate throughout the body to only bind to foreign antigens. This technique is termed negative selection because randomly generated detectors are screened by eliminating the cells which respond to predefined targets rather than keeping them for positive selection. The clonal selection algorithm (CSA) uses the idea that only those B cells that recognize the antigens are selected to proliferate. This selection is subjected to an affinity maturation process which improves the B cells’ affinity to the selective antigens. The affinity is the binding fitness between antigens and antibodies, and its measurement could be done using Hamming distance with binary encodings or Euclidian distance with real valued encodings.

The random generation and mutation processes in CSA has similarities to genetic algorithms (GAs). However, there are several distinct differences between GAs and clonal selection: (1) the population size in clonal selection is adjustable while GAs usually use a fixed total number; (2) clonal selection does not use a crossover operation which is used in GA; and (3) clonal selection can solve and maintain multiple local optima solutions, whereas GAs perform a global optimization. In our work, we used a CSA rather than a GA to detect the arc objects reliably because the CSA performs a multimodal optimization process rather than a unimodal or global optimization, and doesn’t suffer the problem of a single detector taking over the population. Castro et al. [31] proposed the clonal selection algorithm (CSA) or later known as CLONALG. CLONALG is based on clonal selection and affinity maturation principles. In CLONALG, one generation would include the initiation of candidate solutions, selection, cloning, mutation, reselection and population replacement. The authors showed that the CSA can find a set of local optima solutions. For the GA, all candidate solutions would converge to the best solution.

Isa et al. proposed to explore the potential of AIS for shape detection using the clonal selection algorithm [32]. Their algorithm works at the image frame level instead of at the pixel level. The antigens and antibodies are designed as 10-by-10 images using a binary string representation. This makes it not very practical to process normal resolution images. The work by Lu et al. proposed a multi-circle detection approach using AIS for a bladder cancer diagnosis application using a real-valued representation [33]. A recent work by Cuevas et al. proposed a circle detection method based on clonal selection [34]. The circle candidates were designed as antibodies using three circumference points with binary encoding, and real existing circles were treated as antigens. A fitness function was designed to evaluate the match between candidate circles and real circle pixels. Many successful examples of AIS for shape detection have strong connections with the clonal selection algorithm. The difficult part of the implementation is representing the data and effectively extracting the shape features. In Section 3, a new real-valued arc shape detection method will be proposed, and compared with the approaches by Lu et al. and Cuevas et al. The next section gives some further details on the clonal selection approach.

#### 2.2.1. Clonal Selection Algorithm

The clonal selection algorithm (CSA) is inspired from immune systems where only antibodies which have the capability to recognize antigens (i.e., non-self cells) will be selected to proliferate by cloning. The CSA uses five underlying principles [31,32,33,34,35]: (1) maintenance of functionally disconnected memory cells; (2) selection and cloning of stimulated antibodies; (3) suppression of non-stimulated cells; (4) affinity maturation and re-selection of clones with highest affinities; and (5) mutation rate proportional to antibodies affinities. In this paper, the antigen model refers to the optimization problem for multiple arcs detection from the edge map, particularly for grape berry images. The antibody model is a representation of a candidate solution (e.g., prototype arc) for an antigen. Examples of antibodies in CSA are B-cells, T-cells and antigen-specific lymphocytes. CSA uses a selective mechanism to give antibodies (i.e., the candidate solutions) which can recognize an antigen longer holding life spans. These cells are known as memory cells. CSA implements the mechanism by storing the global and local optima of the objective function measurement. The remainder of this section gives some basic definitions of CSA operators, followed by a step-by-step description of the CSA algorithm with adaptations for our berry detection problem.

#### 2.2.2. Definitions

The notation uses boldfaced capital letters to indicate matrices and boldfaced small letters to indicate vectors.

- Antigen: optimization problem and its constraints (multiple arc detection from edge map).
- Antibody: candidate solutions (arc candidates).
- Affinity: objective function measurement for an antibody (arc matching with points on edge map).

The antibody character string **d** denotes the coding of variable vector **x** as **d** = *encode*(**x**) and **x** denotes the decoding of antibody **d** as **x** = *decode*(**d**). The set **I** denotes the antibody space and the antibody population space can be defined as:

(1) $$I^{m}\;=\;\{D:D\;=\;\left(d_{1};d_{2};\ldots .\;;d_{m}\right);\;d_{k}\in I;\;1\;\leq \;k\;\leq \;m$$

where *m* a positive integer denotes the size of the antibody population **D** = {**d**_1_, **d**_2_, …, **d***_m_*} which is an *m*-dimensional group of antibody **d** contained within the antibody space **I**.

#### 2.2.3. CSA Operators

CSA uses three different operators: (1) clonal proliferation operator ($T_{p}^{C}$); (2) affinity maturation operator ($T_{M}^{A}$); and (3) clonal selection operator ($T_{S}^{C}$) [34]. **A**(*k*) is the antibody population at time *k* representing the set of antibodies **a** such that **A**(*k*) = {**a**_1_(*k*), **a**_2_(*k*), …, **a***_n_*(*k*)}. The evolution of CSA can be described as:

(2) $$A\left(k\right)\overset{T_{p}^{C}}{\to }Y\left(k\right)\overset{T_{M}^{A}}{\to }Z\left(k\right)\cup A\left(k\right)\overset{T_{S}^{C}}{\to }A\left(k+1\right)$$

(1) Clonal proliferation operator ($T_{p}^{C}$)

Define:

(3) $$Y\left(k\right)=T_{p}^{C}\left(A\left(k\right)\right)=\left[T_{p}^{C}\left(a_{1}\left(k\right)\right);\;T_{p}^{C}\left(a_{2}\left(k\right)\right);\ldots ..;\;T_{p}^{C}\left(a_{n}\left(k\right)\right)\right]$$

where $Y\left(k\right)=T_{p}^{C}\left(A\left(k\right)\right)=e_{i}\cdot a_{i}\left(k\right),\;i\;=\;1,\;2,\;\ldots ,\;n,$ and where **e***_i_* denotes a *q_i_*-dimensional identity column vector. In this work, *q_i_* is calculated as:

(4) $$q_{i}\left(k\right)=round\;N_{c}\cdot \frac{F\left(a_{i}\left(k\right)\right)}{\sum_{j=1}^{n}\left(a_{j}\left(k\right)\right)},\;i=1;2;\ldots n$$

where *N_c_* denotes the clonal size and the function *round* (*x*) returns *x* as the least integer bigger than *x*. The value of *q_i_*(*k*) is proportional to the value of *F*(**a***_i_*(*k*)). After clonal proliferation, the population becomes:

**Y***(k)*={**Y**_1_*(k),***Y**_2_*(k)*,…,**Y**_n_*(k*)} (5)

where $Y_{i}\left(k\right)=\left\{y_{ij}\left(k\right)\right\}=\left\{y_{i1}\left(k\right);y_{i2}\left(k\right);\ldots ;\;y_{iq_{i}}\left(k\right)\right\}$, $y_{ij}\left(k\right)=a_{1}\left(k\right),\;j=1,2,\;\ldots ,\;q_{i},\;i=1,\;2,\;\ldots n$.

(2) Affinity maturation operator ($T_{M}^{A}$)

The affinity maturation operator ($T_{M}^{A}$) models the process of hypermutation in the immune system and introduces random changes into the antibodies with the view that the changes may lead to increasing the affinity rate. This process is performed by the $T_{M}^{A}$ operator applied to the population **Y**(*k*) by clonal proliferation i.e., $Z\left(k\right)=T_{M}^{A}\left(Y\left(k\right)\right)$. The mutation rate (α) is proportional to the affinities of the antibodies towards the antigens, and is calculated using the equation $\alpha =e^{-\rho \cdot F\left(ab\right)}$ where *F* denotes the objective function of the antibody normalized between 0 and 1. The parameter *ρ* denotes the fixed step and is used to modify the shape of the mutation rate as proposed by Cutello et al. [36]. For binary encoding, the mutation operation can be implemented by replacing each gene within an antibody by its opposite (i.e., 0→1 or 1→0). In this paper which uses a real-valued representation, the mutation is implemented by using a different random starting point and intervals on the encoded antibody string. Following the affinity maturation operation, the population becomes:

**Z***(k)*={**Z**_1_*(k),***Z**_2_*(k)*,…,**Z**_n_*(k*)} (6)

where $Z_{i}\left(k\right)=\left\{z_{ij}\left(k\right)\right\}=\left\{z_{i1}\left(k\right);z_{i2}\left(k\right);\ldots ;\;z_{iq_{i}}\left(k\right)\right\}$, $z_{ij}\left(k\right)=T_{M}^{A}(y_{ij}\left(k\right)),\;j=1,2,\;\ldots ,\;q_{i},\;i=1,\;2,\;\ldots n)$.

(3) Clonal selection operator ($T_{S}^{C}$)

Define *∀i = 1,2,…,n*, **b***_i_*(*k*) ∈ **Z***_i_*(*k*) as the antibody with the highest affinity in **Z***_i_*(*k*), then $T_{S}^{C}$ is defined as:

(7) $$T_{S}^{C}(Z_{i}\left(k\right)\cup a_{i}\left(k\right)=\{\begin{array}{c} b_{i}\left(k\right)\;if\;F\left(a_{i}\left(k\right)\right)<F\left(b_{i}\left(k\right)\right)\\ a_{i}\left(k\right)\;if\;F\left(a_{i}\left(k\right)\right)\geq F\left(b_{i}\left(k\right)\right) \end{array}$$

where *i* = 1, 2, …, *n*.

#### 2.2.4. CSA Algorithm Steps

**Figure 2:**
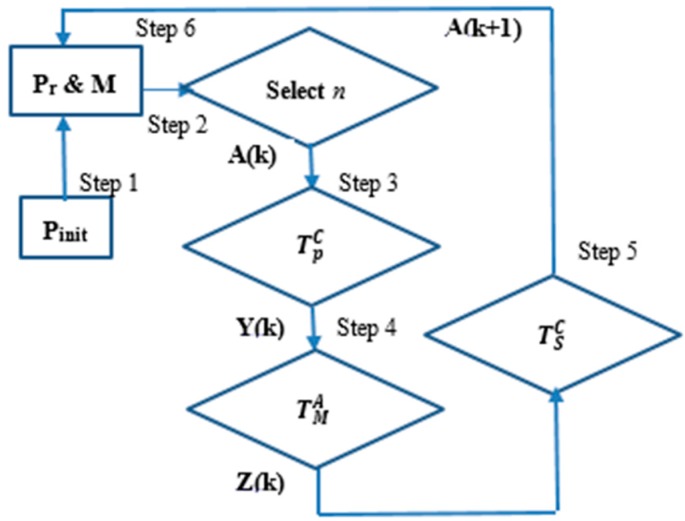
Overview of the clonal selection algorithm.

Figure 2 shows an overview of the CSA using the three different operators. The CSA algorithm is as follows:

**Step** **1:** Initialize a random population (*P*_init_) and a set *h* = *P*_r_ + *n* of candidate solutions of subsets of memory cells (**M**) which is added to the remaining population (*P*_r_). The total population is then given by **P**_T_ = *P*_r_ + **M** where **M** denotes storage for the *n* memory cells.
**Step** **2:** Select the *n* best candidates of population **P**_T_ in order to build **A**(*k*) based on affinity measure.
**Step** **3:** Reproduce ($T_{p}^{C}$) the population **A**(*k*) in proportion to affinity with respect to the antigen and generate a population of clones **Y**(*k*).
**Step** **4:** Mutate ($T_{M}^{A}$) the population **Y**(*k*) of clones based on affinity between antibody and the antigen following Equation (7).
**Step** **5:** Re-select ($T_{S}^{C}$) the best individuals from **Z**(*k*) and **A**(*k*) to give a new memory set **M** = **A**(*k* + 1).
**Step** **6:** Add random *P*_r_ antibodies to the new memory cells **M** to build **P**_T_.
**Step** **7:** Return to Step (2) if the finishing criterion has not been reached.

The antibody addition in Step (6) gives diversity to the population and the CSA can avoid being trapped into local minima solutions. This feature of CSA is in contrast to genetic algorithms which tends to bias the population towards only reaching the best candidate solution. Therefore, the CSA can effectively handle multimodal optimization tasks [37,38]. CSA does not use a crossover operation for population management and instead uses a direct searching algorithm. Only the cloning and hypermutation of individuals are needed to use affinity as the selection mechanism. In this paper, the CSA is used to find the parameters of the multiple arc objects that better corresponds to the actual arc pixels in the edge map.

## 3. Multi-Arc Detection based on Artificial Immune System for Berry Images

The shape detection task continues to be a very important topic in the visual/image processing field, with many applications in the real world. A useful shape detection application is for circular/ring detection. The circular Hough transform has been the most popular for this application, but suffers from high computational load and storage requirements. This section describes our proposed multi-arc detection approach for grape berry images consisting of three stages: (i) Pre-processing to obtain an edge map; (ii) Real-value AIS using the CSA to detect the initial arcs reliably and with good precision; and (iii) Arc extension to obtain the final arcs detected and circles in the grape images for the berry detection viticulture application.

### 3.1. Pre-Processing

**Figure 3:**
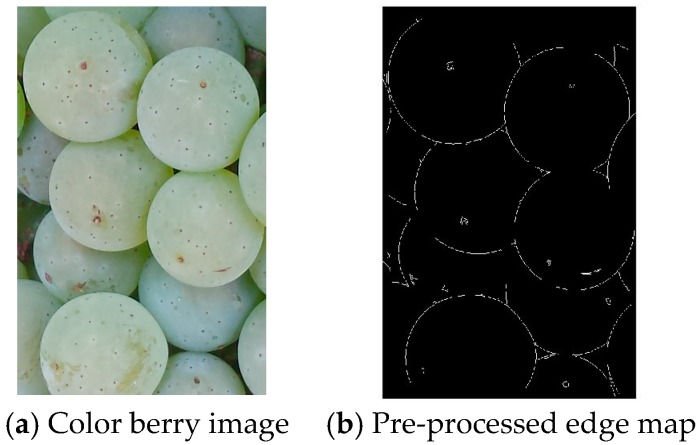
Berry image and its pre-processed edge map.

The input image is first pre-processed to obtain an input edge map. Our approach uses edge detection rather than color-based segmentation because the edge information is more resistant to the range of illumination variations found in the grape berry images. Another reason for not using the color information was to reduce the memory and complexity requirements for the smartphone implementation. Each color berry image (RGB) was first converted to the HSV color space, and the intensity component was used for edge processing. We used the Sobel edge detector followed by a noise filtering process. Other edge detectors were also investigated (such as Canny). By visual inspection of the quality of the filtered images, the Sobel detector was found to give good results for our berry detection applications. Next, a morphological thinning operation was employed to reduce the edge segments to have a single pixel width. Figure 3a,b show a sample berry image and the pre-processed edge map.

### 3.2. Real Value Arc Detection with Artificial Immune System

**Figure 4:**
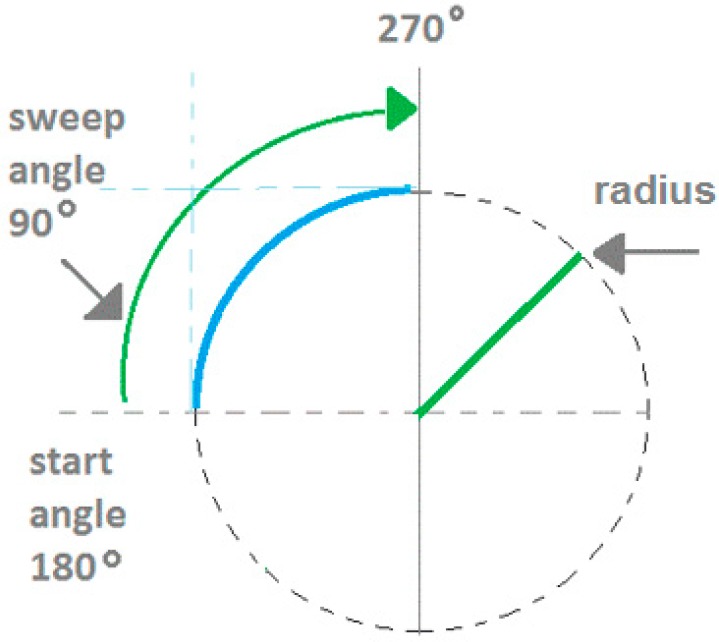
Memory cell representation for arcs.

The pre-processed edge map is used as input into the CSA stage which gives the arcs detected as outputs. The arc edges are modelled as antigens, the arc candidate parameters are modelled as antibodies, and the detected arc parameters are modelled as memory cells. The arcs detected are modelled using five parameters as shown in Figure 4. The parameters are the centre coordinates (*a*,*b*), radius (*r*), start angle (θ), and sweep angle (φ). In the implementation, the antibodies are three random points on the edge map. The real-value representation traces the boundaries of each edge segment using an eight-connectivity chain code. Each chain-indexed edge segment is labelled and becomes an antigen string in the AIS model. The antibody in this model utilizes a three-point representation. A first or starting point is selected along the antigen string. Then, a further two points are selected along regular intervals. The three-point representation has been used for circle representation for GA-based shape detection [39]. Using the three-point representation, an unknown circle can be uniquely identified by storing three parameters (centre coordinates (*x*,*y*), and radius *r*). Parameter values like the radius are obtained as a result of the three-point representation, and does not need to be pre-specified. The arc representation extends this by also storing the start angle and sweep angle.

**Figure 5:**
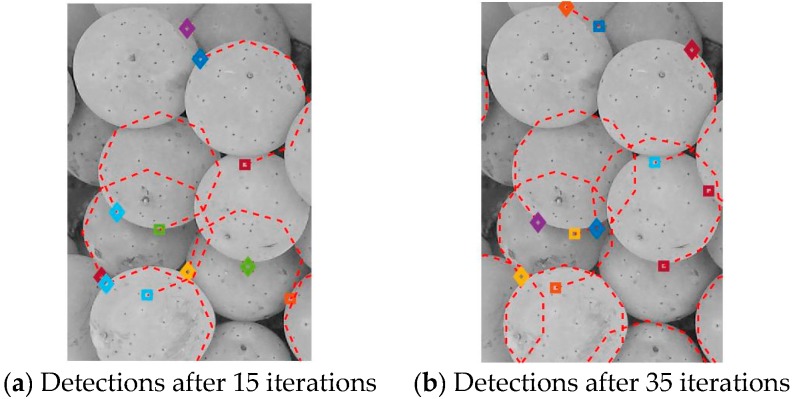
Arc detections from berry image after artificial immune system (AIS) for different iterations.

The antibody is then encoded into a character string consisting of generated pixels along the arc. The affinity ratio or fitness measurement of the antibody towards the antigen is then calculated to evaluate its effectiveness using the Hamming distance between the antigen string and the antibody string divided by the length of the antibody. In this case, the affinity represents a measurement of how many edge pixels are actually located on the derived antibody arc. The antibodies with affinities above a certain threshold are considered to be effective to destroy the antigens and are transformed to become memory cells within the AIS. The antibodies below the threshold undergo a mutation for continuing antigen searching. The mutation of the antibody is performed by using a reselection of the first point and a different interval for the three-point representation. The entire process of generating antibodies to destroy antigens is repeated for a number of iterations. In this work, we used a value of 35 for the number of iterations. The end process of the AIS would be a memory cell list for the arcs detected where each cell item would contain the following pieces of information {centre (*x*,*y*), radius (*r*), start angle (θ), sweep angle (φ), fitness}. The fitness information is also kept to enable the memory cells to be re-ordered for processing in the next stage to obtain the final arcs detected and circles. Figure 5a,b show the arcs detected from the AIS after 15 and 35 iterations respectively.

The works by Lu et al. [33] and Cuevas et al. [34] use AIS techniques to detect full circle objects. As mentioned in the Introduction, after pre-processing, our observation is that some potential arc/circle pixels may be lost in the berry edge map. Our work differs from the existing AIS works in that we focus the AIS optimization on initially detecting strong arcs, which are then extended to form the final circle and arc objects. The work by Rabatel and Guizard [40] proposed using a uniform orange background to capture the berry images using a custom-designed image acquisition tool. In this case, the berry contours would be well-captured, and the circular objects could be directly detected from the edge image. To perform the image acquisition, the berry images were captured in natural environmental conditions (using a smartphone) without specifying a uniform background and with different illuminations and containing other objects (e.g., leaves, stems, etc.). The images were initially captured in the RGB color space in full resolution (i.e., no compression) before conversion to grayscale to perform the arc/circle detection task. The edge map may contain many more spurious points for the object detection task. Other methods may be useful for images taken in constrained environments. However, as discussed in Section 4, our method works reliably for berry images captured in natural conditions. A further point to mention is that some of the grape berries have a green color very close to the leaves. Thus, the color information was not considered in this work at this time.

### 3.3. Arc Extension in Berry Images

After the AIS stage, the arcs in the berry images have been reliably detected and with good precision as shown in Figure 5. However, as shown in Figure 5, the arcs detected do not fully extend along the contours of the berry images. Berries which are not occluded by other berries can have their arcs fully extended towards becoming a full circle. On the other hand, berries which are partially occluded can only be extended until it touches other circles or berries. The order of processing the arcs in the memory cells is important. We use a combination of the arc ratio (*R*) (i.e., how close the arc is already to becoming a circle) and its fitness (*f*) to calculate a circle fitness (*cf*) as *cf* = β*R* + (1 − *β*)*f* where β is a value between 0.0 and 1.0. The memory cells list was sorted in descending order of the *cf*, and the resulting arcs were processed in sequence and classified into another two lists: (i) List of Circles (LC); and (ii) List of Arcs (LA).

**Figure 6:**
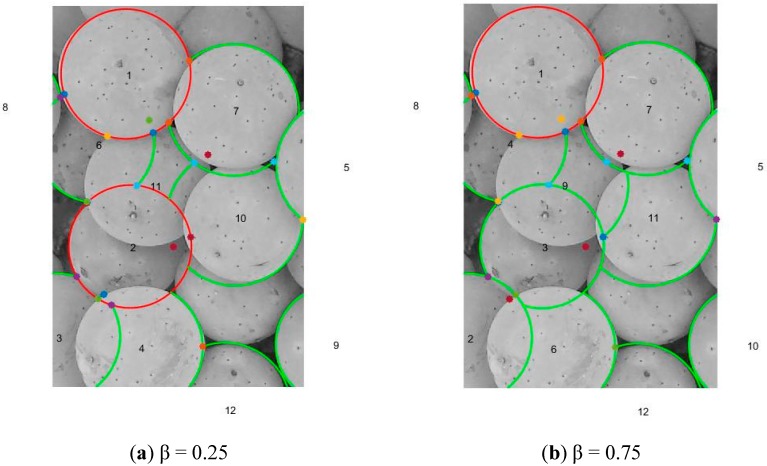
Circle and arc detections from berry image for different β values.

The first entry from the memory cells list is removed. This first entry would have its leftmost and rightmost arcs fully extended and be classified as a circle. The entry is put into the LC. The next entry from the memory cells list is then removed. This entry could either be an arc, if its leftmost or rightmost arcs extends and touches the detected circle in the LC, or it could be a new detected circle if neither of its extended arcs touches the first circle. It is put into the LC if it becomes a new detected circle, else it is put into the LA. This process is repeated for all entries in the memory cell list until the list becomes empty. At the end of this stage, the LCs and LAs contain all the detected circles and arcs respectively. The information in the LCs and LAs can then be used for the berry counting and volume estimation viticulture applications. A point to note is that the outcome from this stage is dependent on the value of β. A higher value of β places emphasis on the arc ratio for the ordering, whereas a lower value places emphasis on the affinity of the arc towards the input edge map. Figure 6a,b show the circles and arcs detected for the values of β = 0.25 and 0.75 respectively.

## 4. Experiments

To verify the performance of the proposed AIS approach, we performed experiments on real-world grape berry images from several vineyards in Australia for comparison with traditional image processing algorithms like the circular Hough transform (CHT) and other well-known circle detection methods. A dataset of 50 berry images from different grape varieties photographed in natural environmental conditions were used for the experiments. The berry images were obtained from domain experts from the National Wine and Grape Industry Centre. Because the CHT can only detect full circles, our comparisons are shown for only the detected circles in the LC list. The performance was calculated using the following three measurements (sensitivity, precision, and Fscore) where TP, FP, FN are the number of true positives, false positives, and false negatives respectively. The TPs show the number of berries detected that were actual berries, the FPs show the number of false berry detections, and the FNs show the number of visible berries that were not detected by the algorithms. The TP, FP and FN parameters were determined by visual inspection in consultation with the domain experts. The CHT gave TP, FN, FP values of 49, 252 and 4 respectively. The RPCD gave TP, FN, FP values of 53, 248 and 3 respectively. The AIS gave TP, FN, FP values of 177, 124 and 22 respectively. Similarly, the recall and precision parameters show the percentage of visible berries detected and the percentage of detections that were berries respectively. The Fscore gives a measure of the trade-off between the precision and recall parameters. The three measurements are calculated as follows:

$$\mathrm{Sensitivity}=\frac{\mathrm{TP}}{\mathrm{TP}+\mathrm{FN}}$$

$$\mathrm{Precision}=\frac{\mathrm{TP}}{\mathrm{TP}+\mathrm{FP}}$$

$$\mathrm{Fscore}=\frac{2\times \mathrm{TP}}{2\times \mathrm{TP}+\mathrm{FP}+\mathrm{FN}}$$

Table 1 shows a comparison of the proposed AIS approach compared with the CHT and the RPCD [41] circle detection method. The RPCD is a parameter-free algorithm which has been shown to be competitive with other circle/ring detection methods like EDCircles [42]. The experiments used a β value of 0.5 to give equal emphasis towards the arc ratio ordering and the affinity of the arc towards the input edge map. In terms of sensitivity, the proposed AIS approach gave a much higher score compared to the other two algorithms. The number of FNs for the AIS were particularly low showing very good detection of berries that were visible. In terms of precision, the AIS gave a slightly lower score than the other two algorithms, with a slightly larger count of FPs. However, using the Fscore as an overall measure to balance between the number of FNs and FPs, it can be seen that the AIS performs very well compared to the CHT and RPCD algorithms. Table 1 also shows that the CHT and RPCD performed similarly for the sensitivity and precision measures, with the RPCD performing slighter better than the CHT for both scores.

## 5. Conclusions

This paper has presented an application of visual information processing (VIP) in viticulture. The paper first gave an overview of the field with examples of current research of VIP in viticulture. Then, a novel visual processing approach for grape berry detection application using a nature-inspired computing approach was presented. The proposed approach considered the multiple arc detection optimization problem and used the clonal selection algorithm to first detect berry arc contours with good precision in correspondence with the input edge map. The arc contours were then extended to cover the full contours of the berries. Experiments performed on grape berry images captured in real-world environmental conditions showed that the approach produces good results compared to other well-known circle detection algorithms. The proposed AIS approach gave a Fscore of 0.71 compared with Fscores of 0.28 and 0.30 for the CHT and a parameter-free circle detection technique (RPCD) respectively showing its potential and usefulness for viticulture berry detection applications. A further advantage of our approach is that it is able to output both circles and arcs using a single processing engine. This feature could also be useful for berry counting and volume estimation viticulture applications.

## Figures and Tables

**Table 1:** Comparison of proposed AIS with other circle detection algorithms for various performance parameters.

	CHT	RPCD [41]	Proposed AIS
Sensitivity	0.1627	0.1760	0.5880
Precision	0.9245	0.9464	0.8894
Fscore	0.2768	0.2969	0.7080
